# Coupling Complete Blood Count and Steroidomics to Track Low Doses Administration of Recombinant Growth Hormone: An Anti-Doping Perspective

**DOI:** 10.3389/fmolb.2021.683675

**Published:** 2021-06-10

**Authors:** Luca Narduzzi, Corinne Buisson, Marie-Line Morvan, Alexandre Marchand, Michel Audran, Yves Le Bouc, Emmanuelle Varlet-Marie, Magnus Ericsson, Bruno Le Bizec, Gaud Dervilly

**Affiliations:** ^1^ Laboratoire D’Etude des Résidus et Contaminants Dans Les Aliments (LABERCA), Oniris, INRΑe, Nantes -44307, France; ^2^ Département des Analyzes, Agence Française de Lutte Contre le Dopage (AFLD), Châtenay-Malabry, French Anti-Doping Agency, Paris, France; ^3^ Sorbonne Université, INSERM, Centre de Recherche St-Antoine (CRSA), Paris, France; ^4^ Institut des Biomolécules Max Mousseron (IBMM), Centre Hospitalier Universitaire de Montpellier, Montpellie, France

**Keywords:** growth hormone, cbc, steroidomics, trend analysis, anti-doping, athlete biological passport 2

## Abstract

Growth Hormone (GH) under its human recombinant homologue (rhGH), may be abused by athletes to take advantage of its well-known anabolic and lipolytic properties; hence it is prohibited in sports by the World Anti-Doping Agency. Due to the rapid turnover of rhGH, anti-doping screening tests have turned to monitor two endocrine biomarkers (IGF-I and P-III-NP), but unfortunately, they show population-wise variability, limiting the identification rate of rhGH users. Previous studies have evidenced the numerous effects of GH on human physiology, especially in hematopoiesis and steroidogenesis. In this work, aiming to discover novel physiological rhGH biomarkers, we analyzed the complete blood count and the steroidomics profile of healthy, physically active, young males treated either with EPO + rhGH or EPO + placebo. The time-trends of these two physiological routes have been analyzed through geometric trajectory analysis (GTA) and OPLS-DA. Individuals supplemented with micro-doses of rhGH exhibited different leukopoietic and steroidal profiles compared to the control population, suggesting a role of the rhGH in both pathways. In the article, hypotheses on the observed differences are discussed according to the most recent literature and compared to results in animal models. The use of leukopoietic and steroidal biomarkers together with endocrine biomarkers (IGF-1 and P-III-NP) allows to correctly classify over 98% of samples with no false positives, miss-classifying only one single sample (false negative) over a total of 56; a promising result, if compared to the current rhGH detection strategies.

## Introduction

Growth hormone is an endocrine factor involved in the regulation of several pathways, like correct body development, metabolic homeostasis, bones and muscles anabolism. It binds directly to its target cells and it also stimulates the release of the insulin-like growth factor 1 (IGF-I), a multi-functional peptide (Bortvedt and Lund, 2012). Receptors for GH and IGF-I are present in several cells types of the body and they are involved in the regulation of several processes (Jones and Clemmons, 1995; Giustina and Veldhuis, 1998). Also, their effect is different depending on the age (Junnila et al., 2013; Blum et al., 2018), ethnicity (Berrigan et al., 2009; Grimberg et al., 2018) and sex (Meinhardt and Ho, 2006) of the subjects.

Growth Hormone is beneficial for athletes due to its well-known anabolic and lipolytic properties. Two main advantages are obtained after abusing rhGH: 1) GH exerts an anabolic effect on muscles, enhancing amino-acids uptake, limiting also proteins breakdown (Bujis et al., 2002; Liu et al., 2006; Velloso, 2008). 2) GH has a lipolytic effect, driving the metabolism toward ketosis.

The rhGH is among the prohibited substances reported in the World Anti-Doping Agency list (WADA, 2019). A first rhGH detection test (Wallace et al., 2001) has been established in 2004, based on the discrepancy between the different natural GH isoforms (22 and 20 kDa) and the singular rhGH isoform (22 kDa). Albeit accurate, the performance of such a test is limited by the rapid turnover of the rhGH (its detectability is in between 12–24 h (Lehtihet et al., 2019; Marchand et al., 2019)). In theory, more effective tests could be developed by monitoring GH bio-markers (Powrie et al., 2007; Ding et al., 2008; Tan et al., 2017; Narduzzi et al., 2020a; Botrè et al., 2020). An official biomarkers-based method has thus been implemented by WADA in 2012, relying on the quantification of IGF-I and of amino-terminal pro-peptide of type III procollagen (P-III-NP) (Erotokritou-Mulligan et al., 2007; Powrie et al., 2007; Holt, 2009; Holt et al., 2015). A score factor (GH-2000 score) based on the concentration of the peptides weighted by athletes’ sex and age (Böhning et al., 2016, 2018), is used to track rhGH administration, against a population-based decision limit. Unfortunately, this test is not efficient in the case of micro-dosing (Lehtihet et al., 2019; Marchand et al., 2019) (consecutive injection of low doses of rhGH) due to the wide response variability to the treatment within the athletes’ population.

A possible strategy to improve the accuracy of such biomarkers-related test is to increase the number of biomarkers involved. In this sense, further peptide markers linked to GH’s cascade pathway have already been proposed by some authors (Holt et al., 2015, 2017; Sieckmann et al., 2020). The extension of the list of biomarkers of GH has already been approached by omics strategies, like proteomics (Ding et al., 2008, 2011; Duran-Ortiz et al., 2017) and metabolomics (Narduzzi et al., 2019; 2020b). Nevertheless, given the high number of physiological processes regulated by GH, there might be further reservoirs of relevant markers that have not been considered yet.

Studies on human and animal physiology indicate that GH is involved also in two very important physiological processes: 1) hematopoiesis (Andreassen et al., 2012) and 2) steroidogenesis (Andreassen et al., 2013). Hematopoiesis is a possible reservoir of rhGH biomarkers; it is well known that rhGH restores normal erythropoiesis in GH-deficient subjects (both in animals and humans) (Muta et al., 1994; Merchav, 1998; Hanley et al., 2005), but its influence on red blood cells seems to be limited in normal subjects (Sieckmann et al., 2020). From the literature, it seems clear that rhGH influences also the production of antibodies (Hattori, 2009) and white blood cells (WBC), with a controversial effect depending on the subject status (Andreassen et al., 2012). To our knowledge, a study on the effects of GH on the whole leukopoiesis and platelets has never been carried out on healthy subjects administered with GH for doping purposes.

With regard to the link between GH and steroids, it is well known that the somatotropic axis is involved in the correct development of the reproductive organs in both males and females. Receptors for GH and IGF-I are indeed present in pituitary gonadotrophs and on testicular Leydig and Sertoli cells (Andreassen et al., 2013); in females, IGF-I is produced from ovarian cells and it is involved in folliculogenesis (Adashi, 1998). GH exerts also an effect on steroidogenesis, increasing the levels of both 17β-estradiol and 17β-testosterone (Andreassen et al., 2013) and determining an accumulation of 17β-testosterone and 5α-dihydro-testosterone in GH-deficient adult males after rhGH administration (Blok et al., 1997). It may consequently be expected that treatment with rhGH would induce an effect on the whole steroidogenic pathway in healthy adults.

The present study aims to identify and discuss the effect of the administration of low doses of rhGH on the hematological and steroidal profiles of young trained volunteers determined by a complete blood count (CBC) and steroidomics analysis, to investigate a wider understanding of the effect of rhGH on human physiology and to identify putative biomarkers. We also combined those highlighted biomarkers with the two biomarkers from the GH-2000 test (IGF-I and P-III-NP) to evaluate the classification accuracy of such a combined statistical model built on the hematopoietic, steroidal and endocrine profiles.

## Materials and Methods

### Human Samples and Study Design

Samples were obtained from a previous study approved by the French ethics committee (N 2016–002742–23) (Marchand et al., 2019): 14 well-trained male volunteers aged between 19–29 years have been split into two equal groups. The first group (EPO, mean age 24 years) received subcutaneous injections of six micro-doses over 2 weeks (three times a week) of recombinant EPO Binocrit (epoetin alpha) at 10 IU/kg combined with placebo (saline solution). The second group (EGH, mean age 23.5 years) received subcutaneous injections of six micro-doses over 2 weeks (once every 2–3 days) of recombinant EPO Binocrit (epoetin alpha) at 10 IU/kg and 2 IU (0.67 mg) of Genotonorm (rhGH). All the volunteers were checked for Ferritin levels and hematological parameters before inclusion and received oral supplement of iron and vitamins during the study. Samples of plasma (5 ml), serum (5 ml) and urine were collected along the whole study. In this study, samples collected at days 0, 4, 11, and 14, (named T0, T4, T11, T14) were analyzed.

**FIGURE 1:**
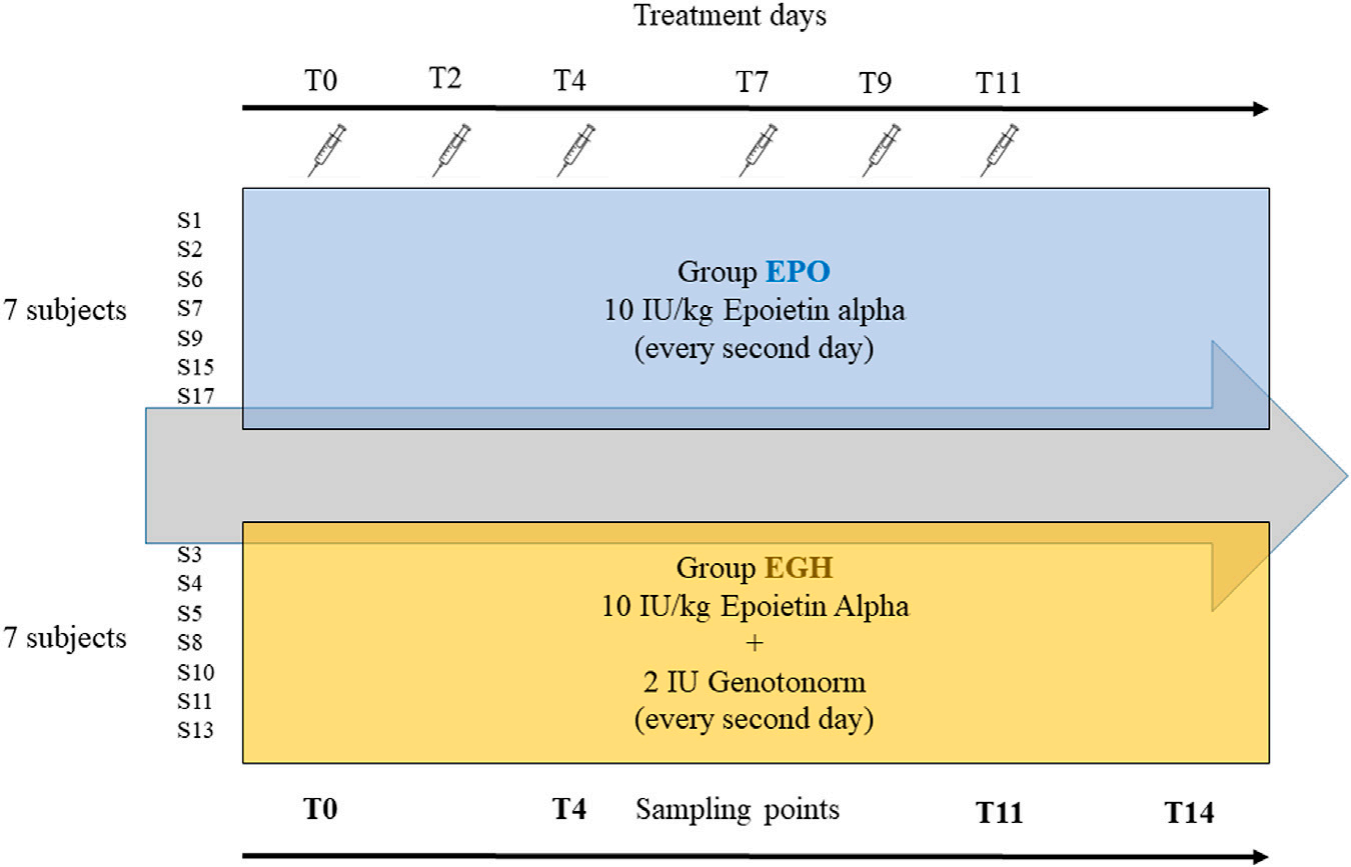
The design of the experiment.

The samples analyzed in this study are from the study of Marchand et al. (Marchand et al., 2019). In their experiment, the subjects have been treated either with EPO or EPO + rhGH to study the synergic effect of GH and EPO on human physiology and their performance. All subjects have been administered with EPO while the “case” group has been treated also with rhGH. A control (placebo) group is missing. In our analysis, the 14 subjects (out of 16) have been analyzed along four time-points (Figure 1).

### Chemicals and Reagents

The purified sulfatase (*Helix pomatia* S9626) and β-glucuronidase (*Patella vulgata* G8132) MSTFΑ, TMIS, DTE, tert-butylmethylether (MTBE), pentane, hexane, ethyl-acetate, ethyl-ether, methanol, dichloromethane and NH_4_I were purchased from SIGMA Aldrich (St. Quentin Fallavier, France). The Bond Elut C18 500 mg-10 cc cartridges were from Chromoptic (Courtaboeuf, France). Deionized water was produced using an ultra-pure water system (Milli-Q, Millipore Darmstadt Germany). IGF-I was from R and D Systems (Bio-Techne France, Lille, France) and P-III-NP (calibrator from the ELF™ blood test) was from Siemens Heatlthineers (Saint-Denis, France).

### The Complete Blood Count (CBC) and the Hematological Module of the Athletes Biological Passport (ABP)

In a classical CBC performed by automated blood analyzers, 33 different blood parameters are counted, representing the three main blood constituents: red blood cells, white blood cells and platelets. In this experiment, all of them have been considered as targets of our analysis. Just for comparison, the hematological module of the ABP (TD2019BAR) consists only of 14 blood parameters (mostly erythropoietic parameters) selected among the original 33. Full blood samples in EDTA tubes (BD Vacutainer®, BD, Le Pont de Claix, France) were analyzed for full blood counts on an automated hematology analyzer, Sysmex XT 2000i (Sysmex Corporation, Kobe, Japan). All the hematopoietic parameters obtained from the instrument are reported in Table 1.

**TABLE 1:** The hematologic parameters and the steroids analyzed in this study.

CBC parameters	Steroidomics parameters
White blood cells (WBC)	5β-androstane-3α,17α-diol
Red blood cells (RBC)	5β-androstane-3β,17α-diol
Hemoglobin (HBG)	5α-androstane-3α,17α-diol
Hematocrit (HCT)	5β-androstane-3α,17β-diol
Mean corpuscular volume (MCV)	5β-androstane-3β,17β-diol
Mean corpuscular hemoglobin concentration (MCHC)	5α-androstane-3β,17α-diol
Mean corpuscular hemoglobin total count (MCHT)	5α-androstane-3α,17β-diol
Platelets (PTL)	5α-androstane-3β,17β-diol
Red cell distribution width standard deviation (RCDW_SD)	5β-dihydrotestosterone(5β-DHT)
Red cell distribution width coefficient of variation (RCDW_CV)	5a-Dihydrotestosterone(5α-DHT)
Platelets distribution width (PDW)	Epiandrosterone
Mean platelets volume (MPV)	Androsterone
Plateletcrite (PCT)	Etiocholanolone
Big platelets proportion (P-RGC)	5-Androstene-3α,17β-diol
Neutrophils percentage (NEUT%)	5-Androstene-3β,17α-diol
Neutrophils total count (NEUT/)	5-Androstene-3β,17β-diol
Lymphocytes percentage (LYMPH%)	5β-androstanedione
Lymphocytes total count (LYMPH/)	5α-androstanedione
Monocytes percentage (MONO%)	Dehydro-epi-androsterone(DHEA)
Monocytes total count (MONO/)	4-Androstenedione
Eosinophils percentage (EO%)	17α-testosterone(epi-testosterone)
Eosinophils total count (EO/)	17β-testosterone(Testosterone)
Basophils percentage (BASO%)	17α-estradiol
Basophils total count (BASO/)	Estrone
Immunoglobulins percentage (IG%)	17β-estradiol
Immunoglobulins concentration (IGconc)	Allopregnenolone
Reticulocytes percentage (RET%)	5a-Pregnane-3α,17a-diol-20-one
Reticulocytes total count (RET/)	5a-Pregnane-17a-ol-3,20-dione
Reticulocytes hemoglobin ratio (RET_He)	Pregnenolone
Immature reticulocytes fraction (IRF)	17a-hydroxy-pregnenolone
Reticulocytes low fluorescence ratio (LFR)	17a-hydroxy-progesterone
Reticulocytes medium fluorescence ratio (MFR)	Progesterone
Reticulocytes high fluorescence ratio (HFR)	5a-Pregnane-3,20-dione

### The Steroidomics Profile and the Steroidal Module of the Athlete Biological Passport

The method used for the extraction and derivatization of sex steroids has been developed and described previously (Hennig et al., 2018; O’Shaughnessy et al., 2019; Savchuk et al., 2019). Using this steroidomics method, 33 different steroids have been quantified (Table 1). Just for comparison, the steroidal module of the ABP (TD2018EAAS) consists in the measurement of only six steroids (out of the 33): Androsterone (A), Etiocholanolone (Etio), 5α-Androstane-3α,17β-diol (5αAdiol), 5β-Androstane-3α,17β-diol (5βAdiol), 17β-Testosterone (T), Epitestosterone (E). Both methods analyzed the steroids excreted in urine after deconjugation and derivatization (Buisson et al., 2018).

Briefly, urine samples (50 µL) were first de-conjugated using a mix of sulfatase (Helix pomatia) and *β*-glucuronidase (Patella vulgata) overnight; steroids were extracted with 10 ml of methyl-tert-butyl-ether (MTBE), dried and re-suspended in 200 µL of methanol. The extracts were purified using a SPE-chromP cartridge and eluted with 14 ml of hexane/ether 70/30 and evaporated to dryness under gaseous N_2_. Androgens were further isolated using 1 ml NaOH 1 M in 6 ml of pentane, while Oestrogens were eluted in 0.75 ml of glacial acetic acid and extracted in 6 ml of pentane. Both androgens and oestrogens were further purified on a silica SPE column, eluted with hexane/ethyl acetate, dried under gaseous N_2_ and resuspended in 200 µL of methanol and derivatized using MSTFA/TMIS/DTE.

Androgens have been quantified using a Gas Chromatography-Electron Ionisation-Triple Quad-Mass spectrometer (EVOQ GC-TQ, Bruker, Champs sur Marne, France) while oestrogens have been quantified using an Atmospheric Pressure Gas Chromatography-Triple Quad-Mass spectrometer (APGC-XEVO-QqQ-MS, Waters, Wilmslow, United Kingdom). The analytical methods used were the same as O’Shaughnessy et al. (2019).

### IGF-I and P-III-NP Profiling

The longitudinal profiling of the IGF-I and P-III-NP peptides (also known as the endocrine profile of the ABP (Sottas et al., 2010)) has been performed following WADA guidelines for the hGH biomarker test (Marchand et al., 2019; WADA, 2019). The two markers were measured in serum: IGF-I concentration was determined using an IDS-ISYS analyzer with IGF-I assay (IS-3900, ImmunoDiagnostic Systems, Paris, France), and P-III-NP concentration was determined with an ADVIA Centaur CP analyzer using the ADVIA Centaur P-III-NP Test (10492440, Siemens, Saint-Denis, France). For both, 180 μL serum was used. A negative control (serum from a healthy volunteer) and a positive control (serum spiked with IGF-I to obtain a value > 500 ng/ml and serum spiked with P-III-NP to obtain a value > 15 ng/ml) were added to validate the results of each dosage.

### Statistical Analysis

All the statistical analysis has been performed in R environment and SIMCA *p*+ 13.0.2 (Umetrics, UMEÅ, Sweden). The datasets have been subjected to Geomeric Trajectory Analysis (Keun et al., 2004) following the formula of Narduzzi et al. (Narduzzi et al., 2020b).

Thus, a low-level fused (Smilde et al., 2005) dataset has been obtained combining (merging) the steroidomics profile, and the hematological and endocrine modules of the ABP and it has been analyzed using sparse-partial least squares-discriminant analysis [sPLS-DA, mixOmics package (Rohart et al., 2017)]. Orthogonal partial least squares-discriminant analysis (OPLS-DA) models have been obtained using SIMCA-P+ 13.0.2. Validation of the models has been achieved using Monte Carlo Cross Validation [MCCV (Xu and Liang, 2001)]. The data used in this study are available as supplementary file (Supplementary Table S1). The PLS-DA and OPLS-DA have been used because they are multivariate discriminant methods that reduce the dimensions of the data into one or few components (like in PCA) and regress the components vs. the categorical variables of the classes, similar to linear regression analysis. PLS-DA and OPLS-DA are discriminant analysis that are advantageous when the number of variables (the measured parameters) is superior to the number of samples, like in this study.

## Results

### Data Structure and Analytical Approach

The dataset of this experiment was constituted of 14 subjects split into two equal groups of seven subjects each. For every subject, a sample of blood and urine were collected at each time point at days 0, 4, 11, and 14 (4 time-points), for a total of 56 different samples. In total, 33 hematological parameters, 33 steroids and two endocrine peptides were analyzed at each time-point. Five steroids 5β-Androstane-3β,17α-diol, 5β-Androstane-3β,17β-diol, 5-androstene-3α,17β-diol, 5α-Pregnane-3,20-dione, and 17αOH-dihydroprogesterone did not meet the limit of detection and have been discarded from the data analysis, reducing their number to 28.

### Statistical Analysis

**FIGURE 2:**
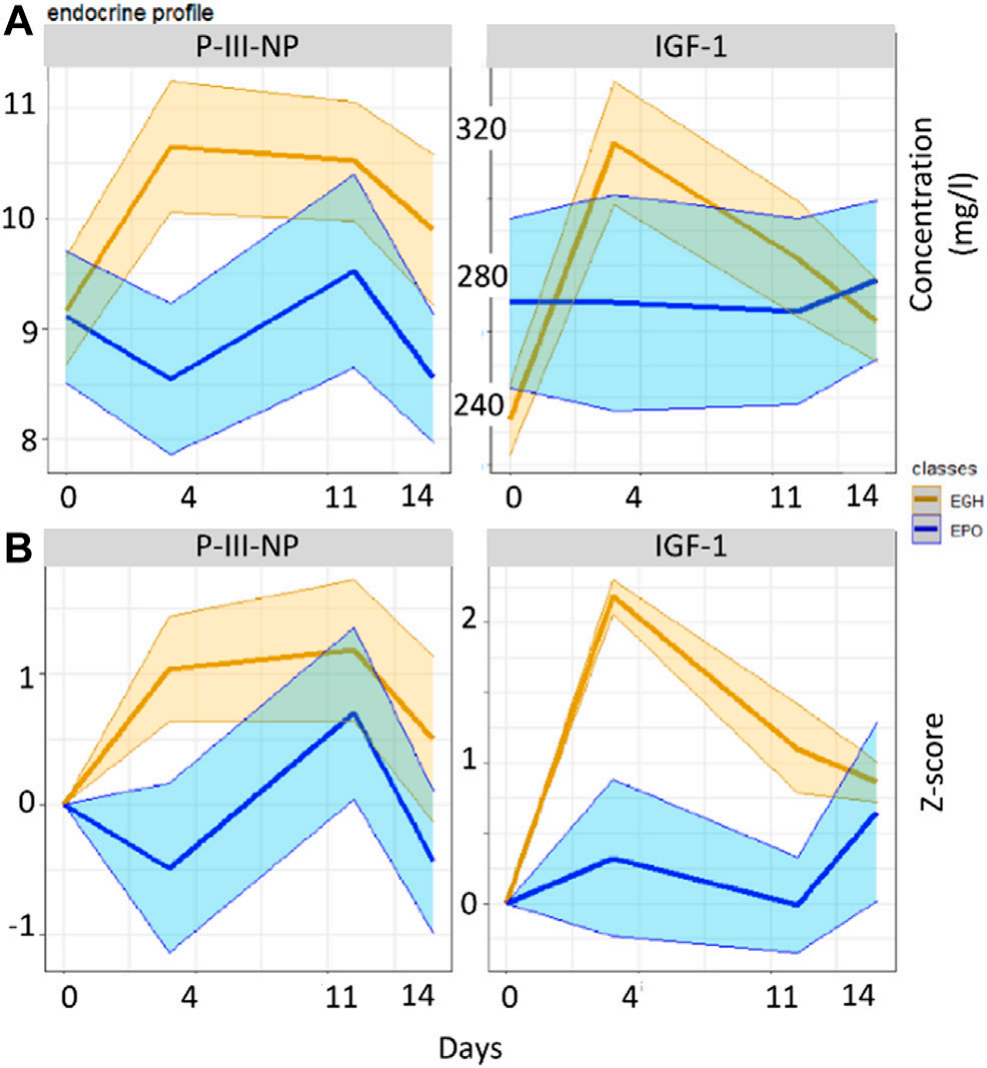
Boxplots reporting the values of the two groups across the various time-points in the original data **(A)** and in the GTA-transformed data **(B)**.

GTA has been applied to our data to take into account the possible lack of homogeneity between the groups. An example of the effect of GTA on inhomogeneous groups is reported in Figure 2 with the measurements of IGF-I and P-III-NP, which are both biomarkers of rhGH administration. When original data are used, P-III-NP shows a better discrimination power than IGF-I between the two groups. This happens because the concentration of IGF-I is higher in the EPO group than the EGH group before the treatments (Figure 2A); but the literature claims that IGF-I is a better marker than P-III-NP, because it is the main target of GH’s cascade pathway and it is accumulated rapidly after rhGH treatment (Holt, 2009). Normalizing the data according to GTA shows a different picture, with IGF-I becoming a better marker than P-III-NP for rhGH treatment (Figure 2B).

GTA transformation has been applied also to all the CBC (*n* = 33) and the steroidomics profiles (*n* = 28). The singular datasets (CBC, steroidomics and endocrine profiles) and the combinations thereof have been evaluated for their classification accuracy and their AUROC (area under the ROC curve) using OPLS-DA. OPLS-DA has been compared to two further models (Random Forest and Elastic Net and it showed better classification accuracy than the latter (data not shown). As shown in Table 2, every profile has some classification property over the samples. The lone endocrine profile (IGF-I + P-III-NP) reaches a classification accuracy at about 75%; an outcome similar to the longitudinal analysis of the GH-2000 score performed by Marchand et al. (Marchand et al., 2019). Both the CBC and the steroidomics profile show a better classification accuracy than the lone endocrine profile (90 and 78%, respectively). The combination thereof reaches 94% while the combination of all three profiles arrives almost to 100% (98%).

**TABLE 2:** The AUROC of classification of the various datasets after 100 Monte Carlo Cross Validations.

	AUROC				Classification accuracy
	Control group	EGH group	EPO group	Overall	Overall
Endocrine (n = 2)	0.7352	0.7840	0.5532	0.69	75%
CBC (n = 33)	0.9630	0.8830	0.7535	0.8665	90%
Steroidomics (n = 28)	0.6063	0.8690	0.7883	0.7533	78%
CBC + steroidomics (n = 61)	0.9546	0.9349	0.8501	0.91	94%
CBC + steroidomics + endocrine (n = 63)	0.9452	0.9740	0.8646	0.93	98%

**FIGURE 3:**
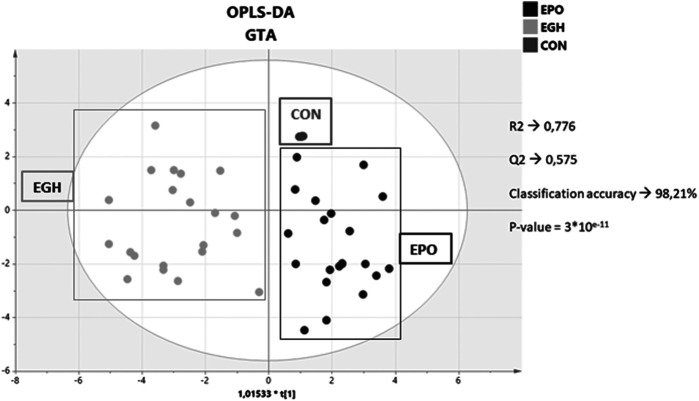
Score plot of the OPLS-DA model on the combined dataset with the hematopoietic, steroidomics and endocrine data.

A score plot of the OPLS-DA of the three combined datasets is reported in Figure 3. As shown, the samples from the two groups are nicely separated. The control group (the samples from the subjects before the treatments) plots altogether in a simple spot. This is an effect of the GTA transformation because the samples before the treatment are used to normalized the data subject-wise. Interestingly, the samples from the EPO treated subjects plot closer to the control group; indeed, the EPO group is more similar to the control group than the EPO + GH group. This means that the combined administration of EPO and GH increases the deviation of the parameters trajectory in comparison to the lone EPO administration (this increase is measurable and it is on average 0.3 standard deviations).

**FIGURE 4:**
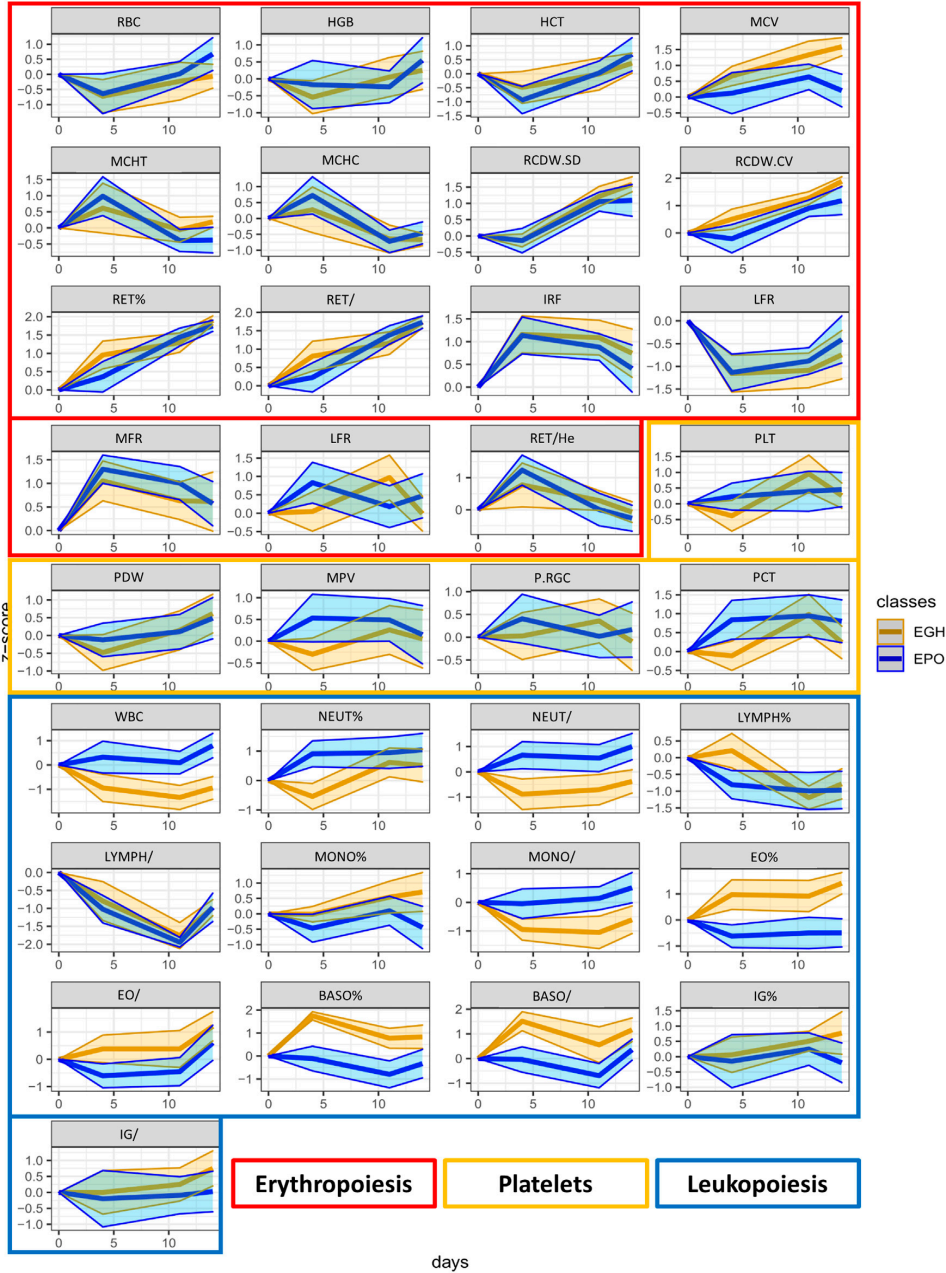
The trend plots of all the hematopoietic parameters measured in our two treated groups (EPO in red and EGH in green). The colored areas represent the trends across the time-points. The area is measured as ±1 standard deviation from the mean value.

**FIGURE 5:**
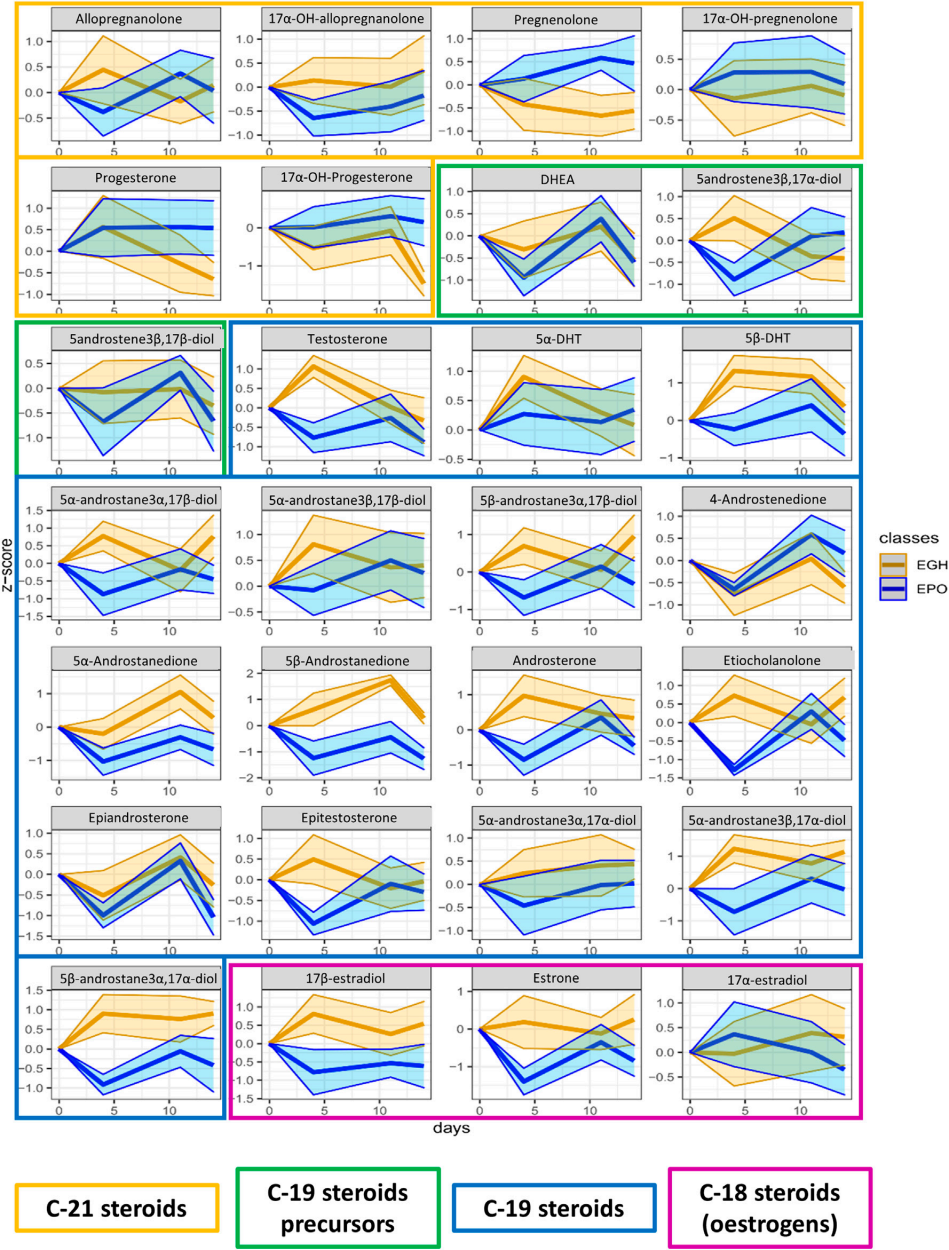
Trend plots of the whole steroidomics profile measured in the urine of the two groups (EPO in red and EGH in green). The colored areas represent the trends across the time-points. The area is measured as ±1 standard deviation from the mean value.

From the OPLS-DA model, a list of putative biomarkers of rhGH administration has been extrapolated and it is now reported in Table 3. The putative biomarkers are ordered according to their importance in the scores’ projection of the OPLS-DA model (variable importance in projection = VIP), which somehow reflects their importance in the correct classification of the samples. As shown, the most important variable in the classification is IGF-I. Unexpectedly, further parameters are in the following positions preceding P-III-NP (which is only in the eighth position). The percentage of the eosinophils and basophils and the counts of the WBC and neutrophils have more discriminant power than P-III-NP, while 5β-Androstanedione, 5β-Androstane-3α-17α-diol, and 17β-estradiol exhibit a VIP value similar to P-III-NP. To better visualize all the changes in the CBC and the steroidomics profile, the GTA transformed data of all the parameters are reported in Figure 4 (complete blood count) and Figure 5 (steroidomics). Regarding the hematopoietic parameters, they could be divided into three groups according to their nature: erythropoiesis, leukopoiesis and platelets count. Despite being one of the main candidate markers to track rhGH in many studies, erythropoiesis did not seem to be affected significantly by rhGH administration. Reticulocytes percentage and count showed a clear rise in both groups (the effect of the EPO treatment) but rhGH did not influence this process significantly. On the other end, leukopoiesis seems to be deeply affected by rhGH administration. The EGH group showed a lower concentration of WBC, neutrophils and monocytes, and interestingly a rise in the percentage of eosinophils and basophils. Immunoglobulins did not show a clear trend in the analysis, but the measured data were always close to zero, due to the low sensitivity of the Sysmex instrument, so the real difference might be hidden behind the measurement error, (i.e. a more specific test would be required for immunoglobulins). No significant difference could be registered for the platelets level, which seemed to stay similar between the two groups.

**TABLE 3:** The putative biomarkers of rhGH administration with the relative Variable Important in Projection (VIP) value.

Parameters	VIP value
IGF1	1.803
Eosinophils%	1.785
Neutrophils concentration	1.63
WBC	1.628
Basophils%	1.538
5β-androstanedione	1.468
5β-androstane-3α-17α-diol	1.439
P-III-NP	1.413
17β-estradiol	1.395
Basophils concentration	1.244
Neutrophils%	1.232
Eosinophils concentration	1.214
Mean globuli volume (MGV)	1.209
Red cell dimension width (RCDW)	1.208
Monocytes concentration	1.189
5α-androstane-3α-17β-diol	1.144
Etiocholanolone	1.135
Pregnenolone	1.108
Androsterone	1.093
5α-androstanedione	1.087
5β-androstane-3α-17β-diol	1.066
Estrone	1.02
5α-androstane-3β-17α-diol	0.996
17β-testosterone	0.962
5β-DHT	0.935

In the steroidomics profile, the situation was different; many of the urinary steroids showed to be affected by rhGH. In particular, 5β-Androstane-3α,17α-diol, 5β-Androstane-3α,17β-diol, 5α-Androstane-3β,17α-diol, 5β-DHT, 5α-DHT, Androsterone, Etiocholanolone, 5-androstene-3β,17α-diol, 5β-Androstanedione, 5α-Androstanedione, DHEA, 5α-Androstane-3α,17β-diol, 5α-Androstane-3β,17β-diol, 17α-testosterone, 17β-testosterone, Estrone, 17β-estradiol, Allopregnanolone, and 17αOH-Allopregnanolone showed higher excretion levels four days after treatment in the EGH group. This difference appeared to be mitigated by day 11. To confirm such finding, we analyzed also the data from the steroidal module of the ABP obtained on the same sample-set in the analysis department of the French anti-doping agency following the WADA recommendations (Buisson et al., 2018; WADA, 2018). The result of the steroidal module confirmed the previous observations, indicating a strong increase at day four and mitigation by day nine (Supplementary Figure S1). Considering all the time points, it is clear from the figure that there is a clear difference in the trends of 5β-androstanedione, 5α-androstanedione, 5β-androstane-3α,17α-diol, and 17β-estradiol between the two groups.

## Discussion

From an anti-doping perspective, it is clear that the hematological and steroidomics profile enhance the odds to track rhGH administration. The subjects of this study were all negative using the GH-2000 score test (one of the official WADA tests) and only 50% of the treated samples could be correctly classified by the longitudinal tracking of the endocrine module of the ABP (Sottas and Vernec, 2012). The endocrine module does not suffer from false-positive classification, so its classification accuracy is 75% (Marchand et al., 2019); higher than the current GH-2000 score, but still rather inaccurate.

The analysis of the CBC and steroidomics profiles through GTA transformation and OPLS-DA increases the chance of having a classification close to 100% (Table 2). The result is very promising and does not require a lot more work in terms of laboratory effort, since the current analysis are already implemented for the ABP. Indeed, the haematological module is only a subset of the whole CBC, while the steroidal module is similar to the steroidomics analysis in terms of sample extraction, preparation and instrumentation used.

The application of GTA transformation has been the key to analyze this kind of data. Indeed, the lack of homogeneity between subjects is common and the response to treatment may vary, especially in the case of pseudo-endogenous substances administered in micro-doses. The use of OPLS-DA is necessary to identify the most promising biomarkers. Nevertheless, once the biomarkers would be confirmed and validated (through further experiments), we do not exclude the possibility to apply a Bayesian model on a score obtained from the multiple biomarkers selected, similar to the ABP score (Sottas et al., 2006).

The results showed that both hematopoietic and steroidogenic processes are affected by rhGH administration even at low doses. Among the various hematologic parameters, leukocytes appear to be the most affected by the treatment. Administration of rhGH induces a general decrease of WBC, and in particular neutrophils and monocytes while increasing the percentage of eosinophils and basophils. A mixed effect on the immune system of rhGH has already been reported from previous research (Meazza et al., 2004). GH exhibits, most likely, an anti-inflammatory effect on the body, but the production of specific immunoglobulins to administered GH induces the production and its binding by specific immunoglobulins (Prader et al., 1964; Roth et al., 1964) in both humans and animals, in particular IgA and IgG (Burton et al., 1991). This might enhance the accumulation of specific WBC like the eosinophils and basophils. Interestingly, specific tests for anti-doping controls based on the detection of specific immunoglobulins binding rhGH have been developed for horses (Bailly-Chouriberry et al., 2008), with a routine method for anti-doping controls in livestock as a result (Rochereau-Roulet et al., 2011). More specific tests might be needed to identify if and what kind of Igs are related to rhGH administration in humans.

Many previous studies focused on the erythropoietic effect of rhGH, following studies indicating a restoring effect of the normal erythropoiesis in GH-deficient subjects treated with rhGH (Muta et al., 1994; Merchav, 1998; Hanley et al., 2005). Our study demonstrated that rhGH does not influence the amount and the type of red blood cells in healthy adults in presence of rhEPO. GH and IGF-I are involved in erythropoiesis regulation (Muta et al., 1994; Merchav, 1998; Hanley et al., 2005), i.e. they are necessary for correct red blood cells homeostasis. However, GH and IGF-I do not enhance erythropoiesis under normal conditions (Sieckmann et al., 2020), nor in presence of rhEPO.

**FIGURE 6:**
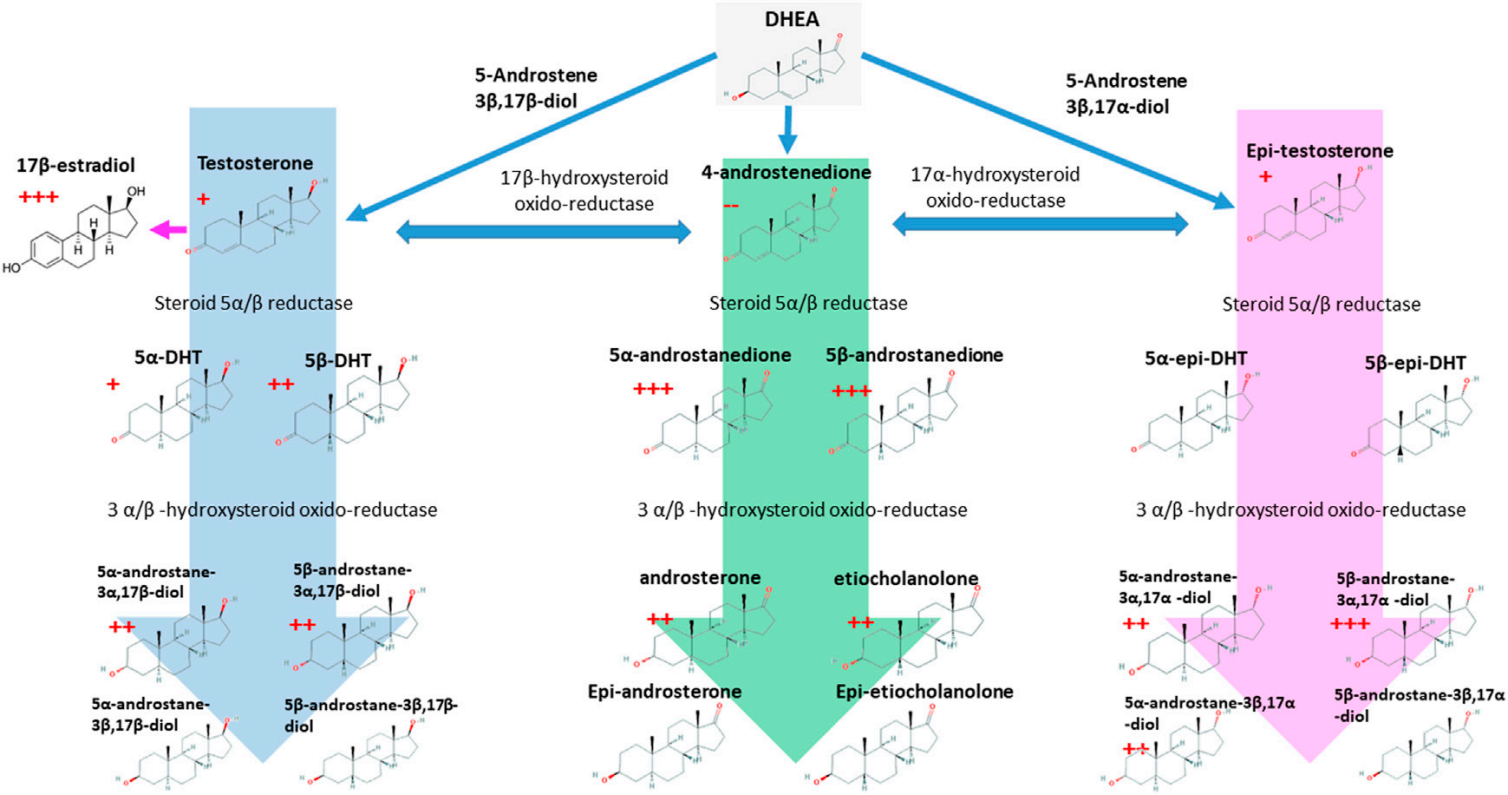
The C-19 steroids pathway and its link with the 17β-estradiol. The red crosses indicated the number of time-points where each molecule resulted to be significantly over-accumulated in rhGH subjects. The red minus indicates the opposite. *p*-value < 0.05.

In the present study, steroidogenesis was observed as impacted by the rhGH treatment. Many of the steroids monitored reacted significantly and presented a profile change to the short-term post-administration, and some of them remained altered across the whole time-course of the experiment. Urinary levels of 5β-androstanedione, 5α-androstanedione, 5β-androstane-3α,17α-diol, and 17β-estradiol were significantly altered by rhGH administration. The precursor, DHEA, is not affected by the treatment, conversely, the rhGH pushes the metabolism toward the excretion of Testosterone, Epitestosterone, the dihydrolated forms (DHTs) and overall the excretion of Androstanes (Figure 6). Interestingly, the concentration of 4-androstenedione decreases, while it increases for its metabolites 5α- and 5β-androstanedione and Androsterone and Etiocholanolone. It may thus be hypothesised that rhGH enhances the activity of the enzymes involved in the reduction of androstenedione (5α- and 5β-reductase particularly), inducing a decrease of its concentration while being actively converted to its reduced counterparts.

The effect of rhGH on 17β-estradiol is obvious in our data; similar results have been already reported for rhGH administration in males (Andreassen et al., 2013). Moreover, during pubertal growth, 17β-estradiol enhances GH release in healthy subjects determining the rapid growth of bones (in association with IGF-I), muscles and the correct development of the reproductive female system (Leung et al., 2004). In our opinion, the effect of rhGH on the whole steroidal profile of healthy adult subjects should be studied more in-depth, as it is a complex, time-dependent process that is still not fully understood.

Some analytical and physiological questions remain. The relationship between rhGH and the hematological and steroidal markers reported here is not completely understood. From an anti-doping perspective, several physiological aspects might act as confounding factors, decreasing the classification properties of the combined model. It poses a question on the importance of the sampling time; sampling just before or right after the exercise may change completely the whole hematological and steroidal pictures. Also, the combined effect of other substances, (i.e. androgenic steroids) might lead to a different steroidomics profile which might be confounding to the statistical classification tools.

Last but not least, confounding factors might have a large effect on this kind of studies and they should be taken into account. To mention some: infections, some concurrent immunity disorders, strenuous exercise, or the interaction with some medicines might deeply affect the WBC counts. Within the steroidal profile, some external factors like stress, excessive exercise, growth and hormonal disorders might deeply affect the whole steroidogenic process. The well-known presence of confounding factors needs to be better evaluated using a wider population of athletes.

## Data Availability

The original contributions presented in the study are included in the article/Supplementary Material, further inquiries can be directed to the corresponding authors.

## Abbreviations

ABP, Athletes Biological Passport; AUROC, Area under the ROC curve; GH, Growth Hormone; GTA, Geomtetric Trajectory Analysis; IGF, Insulin-like Growth Factor; P-III-NP, mino-terminal pro-peptide of procollagen type III; rhGH, recombinant human Growth Hormone; WADA, World Anti-Doping Agency
