# Supplementary figures and images for: Coupling Complete Blood Count and Steroidomics to Track Low Doses Administration of Recombinant Growth Hormone: An Anti-Doping Perspective
Source: Front Mol Biosci. 2021 Jun 10;8:683675. doi: 10.3389/fmolb.2021.683675 (PMC8222787; doi:10.3389/fmolb.2021.683675)

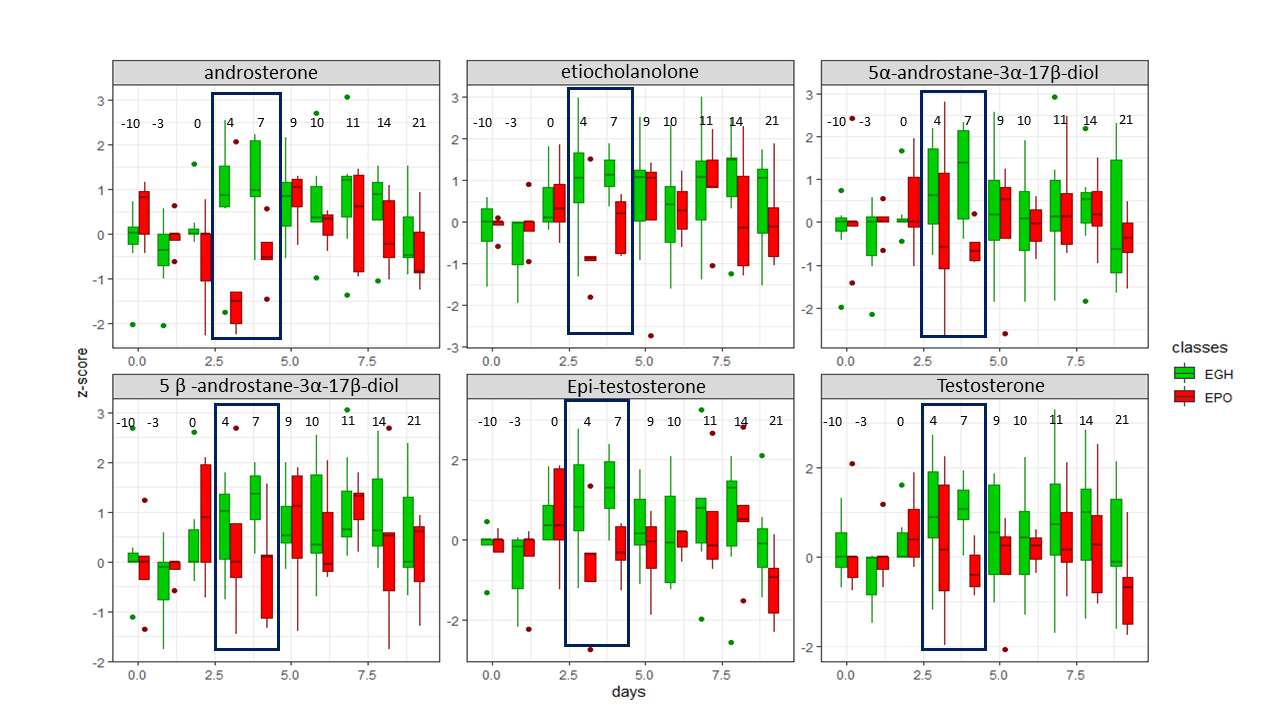

Supplement: Supplementary file 1 [file Image1.TIF]
